# Finding the most appropriate mouse model of juvenile CLN3 (Batten) disease for therapeutic studies: the importance of genetic background and gender

**DOI:** 10.1242/dmm.018804

**Published:** 2015-04

**Authors:** Attila D. Kovács, David A. Pearce

**Affiliations:** 1 Sanford Children’s Health Research Center, Sanford Research, Sioux Falls, SD 57104, USA.; 2 Department of Pediatrics, Sanford School of Medicine, University of South Dakota, Sioux Falls, SD 57104, USA.

**Keywords:** Juvenile neuronal ceroid lipofuscinosis, Batten disease, CLN3, *Cln3*^−/−^ mouse model, *Cln3^Δex7/8^*-knock-in mouse model, 129S6/SvEv, C57BL/6J

## Abstract

Mutations in the *CLN3* gene cause a fatal neurodegenerative disorder: juvenile CLN3 disease, also known as juvenile Batten disease. The two most commonly utilized mouse models of juvenile CLN3 disease are *Cln3*-knockout (*Cln3*^−/−^) and *Cln3^Δex7/8^*-knock-in mice, the latter mimicking the most frequent disease-causing human mutation. To determine which mouse model has the most pronounced neurological phenotypes that can be used as outcome measures for therapeutic studies, we compared the exploratory activity, motor function and depressive-like behavior of 1-, 3- and 6-month-old *Cln3*^−/−^ and *Cln3^Δex7/8^*-knock-in mice on two different genetic backgrounds (129S6/SvEv and C57BL/6J). Although, in many cases, the behavior of *Cln3*^−/−^ and *Cln3^Δex7/8^* mice was similar, we found genetic-background-, gender- and age-dependent differences between the two mouse models. We also observed large differences in the behavior of the 129S6/SvEv and C57BL/6J wild-type strains, which highlights the strong influence that genetic background can have on phenotype. Based on our results, *Cln3*^−/−^ male mice on the 129S6/SvEv genetic background are the most appropriate candidates for therapeutic studies. They exhibit motor deficits at 1 and 6 months of age in the vertical pole test, and they were the only mice to show impaired motor coordination in the rotarod test at both 3 and 6 months. *Cln3*^−/−^ males on the C57BL/6J background and *Cln3^Δex7/8^* males on the 129S6/SvEv background also provide good outcome measures for therapeutic interventions. *Cln3*^−/−^ (C57BL/6J) males had serious difficulties in climbing down (at 1 and 6 months) and turning downward on (at 1, 3 and 6 months) the vertical pole, whereas *Cln3^Δex7/8^* (129S6/SvEv) males climbed down the vertical pole drastically slower than wild-type males at 3 and 6 months of age. Our study demonstrates the importance of testing mouse models on different genetic backgrounds and comparing males and females in order to find the most appropriate disease model for therapeutic studies.

## INTRODUCTION

Neuronal ceroid lipofuscinoses, also known as Batten disease, are a group of recessively inherited, fatal lysosomal storage disorders characterized by progressive neurodegeneration and the intracellular accumulation of autofluorescent lipopigment (Goebel and Wisniewski, 2004). Batten disease is a rare disease and mostly affects children. Based primarily on the age of onset, Batten disease is classified into six clinical forms: congenital, infantile, late infantile, juvenile, Northern epilepsy and adult (Kufs disease) (Schulz et al., 2013). Vision loss, seizures, and progressive cognitive and motor decline are the common symptoms, and the neurodegeneration ultimately leads to early death. The different forms of Batten disease are currently associated with mutations in 14 genes (Warrier et al., 2013).

Mutations of the *CLN3* gene cause the majority of the most prevalent, juvenile-onset, form of Batten disease, and this disorder is now called juvenile CLN3 disease to clearly identify the genetic cause and clinical form (International Batten Disease Consortium, 1995; Williams and Mole, 2012). The disease begins between 4 and 10 years of age, and reaches its terminal stage in the late teens or early 20s. *CLN3* encodes a putative lysosomal/endosomal transmembrane protein, but the exact function of CLN3 and why *CLN3* mutations cause selective neurodegeneration are still unknown. No specific treatment is currently available that could halt or slow the progression of the disease.

The two most commonly utilized mouse models of juvenile CLN3 disease are *Cln3*-knockout (*Cln3*^−/−^) and *Cln3^Δex7/8^*-knock-in mice. The *Cln3^Δex7/8^* model was generated by targeted recombination to remove exons 7 and 8 from the endogenous *Cln3* gene. This created a ‘knock-in’ of exon 7/8-deleted *Cln3*, to mimic the most frequent disease-causing human mutation (Cotman et al., 2002). The *Cln3*^−/−^ model was created by replacing the start codon and the first six exons with a *neo* cassette (Mitchison et al., 1999); *Cln3*^−/−^ mice on the 129S6/SvEv genetic background have been extensively studied (Chan et al., 2009; Kovács and Pearce, 2008; Kovács et al., 2012; Kovács et al., 2011; Kovács et al., 2006; Osório et al., 2009; Pears et al., 2005; Pontikis et al., 2004; Seehafer et al., 2011; Seigel et al., 2002; Weimer et al., 2007; Weimer et al., 2009; Weimer et al., 2006). The *Cln3^Δex7/8^*-knock-in mouse model was generated and initially characterized on a mixed 129S6/SvEv × CD1 genetic background (Cotman et al., 2002; Pontikis et al., 2005). In subsequent studies, *Cln3^Δex7/8^* mice inbred either on the C57BL/6J or C57BL/6N genetic background were used (Finn et al., 2011; Osório et al., 2009; Staropoli et al., 2012). Both *Cln3*^−/−^ and *Cln3^Δex7/8^* mice showed characteristic features of the human disease, including intracellular accumulation of autofluorescent storage material (Cotman et al., 2002; Mitchison et al., 1999), astrocytosis and microglial activation (Cotman et al., 2002; Pontikis et al., 2004; Pontikis et al., 2005; Weimer et al., 2009), neuronal loss (Pontikis et al., 2004; Pontikis et al., 2005; Weimer et al., 2007; Weimer et al., 2009; Weimer et al., 2006), and neurobehavioral abnormalities (Cotman et al., 2002; Finn et al., 2011; Kovács et al., 2011; Kovács et al., 2006; Osório et al., 2009; Staropoli et al., 2012; Weimer et al., 2009). Striking differences exist, however, between the two mouse models in the age-dependency, extent and regional specificity of glial activation and neuronal loss (Pontikis et al., 2004; Pontikis et al., 2005), as well as in the onset and progression of neurological deficits (Osório et al., 2009; Staropoli et al., 2012; Weimer et al., 2009). These differences could be the result of the different genetic backgrounds and/or a residual function or toxic effect of the mutant CLN3 in *Cln3^Δex7/8^* mice.

**TRANSLATIONAL IMPACT**

**Clinical issue**

Batten disease, also known as neuronal ceroid lipofuscinoses, is a group of rare fatal lysosomal storage disorders characterized by progressive neurodegeneration. The disease mostly affects children. Mutations of the *CLN3* gene cause the majority of the most prevalent, juvenile-onset, form of the disease, which presents between 4 and 10 years of age and reaches its terminal stage in the late teens or early 20s. No specific treatment is currently available to halt or slow disease progression. To study the pathomechanisms of juvenile Batten disease and develop therapeutic approaches, various mouse models have been developed. It has not been determined, however, which mouse model on which genetic background is the most suitable for therapeutic studies.

**Results**

The two most commonly utilized mouse models of juvenile Batten disease are *Cln3*-knockout (*Cln3*^−/−^) and *Cln3^Δex7/8^*-knock-in mice, the latter mimicking the most frequent disease-causing human mutation. The two mouse models, however, are on different genetic backgrounds. To determine which mouse model on which genetic background has the most pronounced neurological phenotypes that can be used as outcome measures for therapeutic studies, the authors compared the exploratory activity, motor function and depressive-like behavior of 1-, 3- and 6-month-old *Cln3*^−/−^ and *Cln3^Δex7/8^*-knock-in mice on two different genetic backgrounds (129S6/SvEv and C57BL/6J). The authors found genetic-background-, gender- and age-dependent differences between the two mouse models. Large differences in the behavior of the 129S6/SvEv and C57BL/6J wild-type strains were also observed, highlighting the strong influence that genetic background can have on phenotype. Based on the authors’ results, *Cln3*^−/−^ male mice on the 129S6/SvEv genetic background are the most appropriate candidates for therapeutic studies. They exhibited motor deficits at 1 and 6 months of age in the vertical pole test, and were the only mice to show impaired motor coordination in the rotarod test at both 3 and 6 months.

**Implications and future directions**

This study provides the first behavioral comparison of *Cln3*^−/−^ and *Cln3^Δex7/8^*-knock-in mice on identical genetic backgrounds. The differences observed reveal that, unlike as previously thought, *Cln3^Δex7/8^* mice might not be true nulls but express residual truncated CLN3. The study demonstrates the importance of testing mouse models on different genetic backgrounds and comparing males and females in order to find the most appropriate disease model for therapeutic studies. Using the most suitable mouse model of a human disease in preclinical drug testing greatly enhances the chance of advancing to successful clinical trials.

To determine which mouse model of juvenile CLN3 disease has the most pronounced behavioral phenotypes at different ages and, therefore, is the most suitable for therapeutic experiments, we compared the exploratory activity, motor skills and depressive-like behavior of 1-, 3- and 6-month-old *Cln3*^−/−^ and *Cln3^Δex7/8^*-knock-in mice. Because the genetic background can have a strong influence on the phenotypes of transgenic mice (Bilovocky et al., 2003; Duysen and Lockridge, 2006; Kelly et al., 1998; Lariviere et al., 2001; Lloret et al., 2006; Magara et al., 1999; Mahajan et al., 2004; Nguyen et al., 1997; Tang et al., 2003; Yang et al., 2005) and gender-specific differences exist in disease severity and progression (Ober et al., 2008), we compared *Cln3*^−/−^ and *Cln3^Δex7/8^* mice on two different genetic backgrounds (129S6/SvEv and C57BL/6J), males and females separately.

## RESULTS

### *Cln3*^−/−^ and *Cln3^Δex7/8^*-knock-in mice have genetic-background-, gender- and age-dependent differences in exploratory activity

**Fig. 1.**
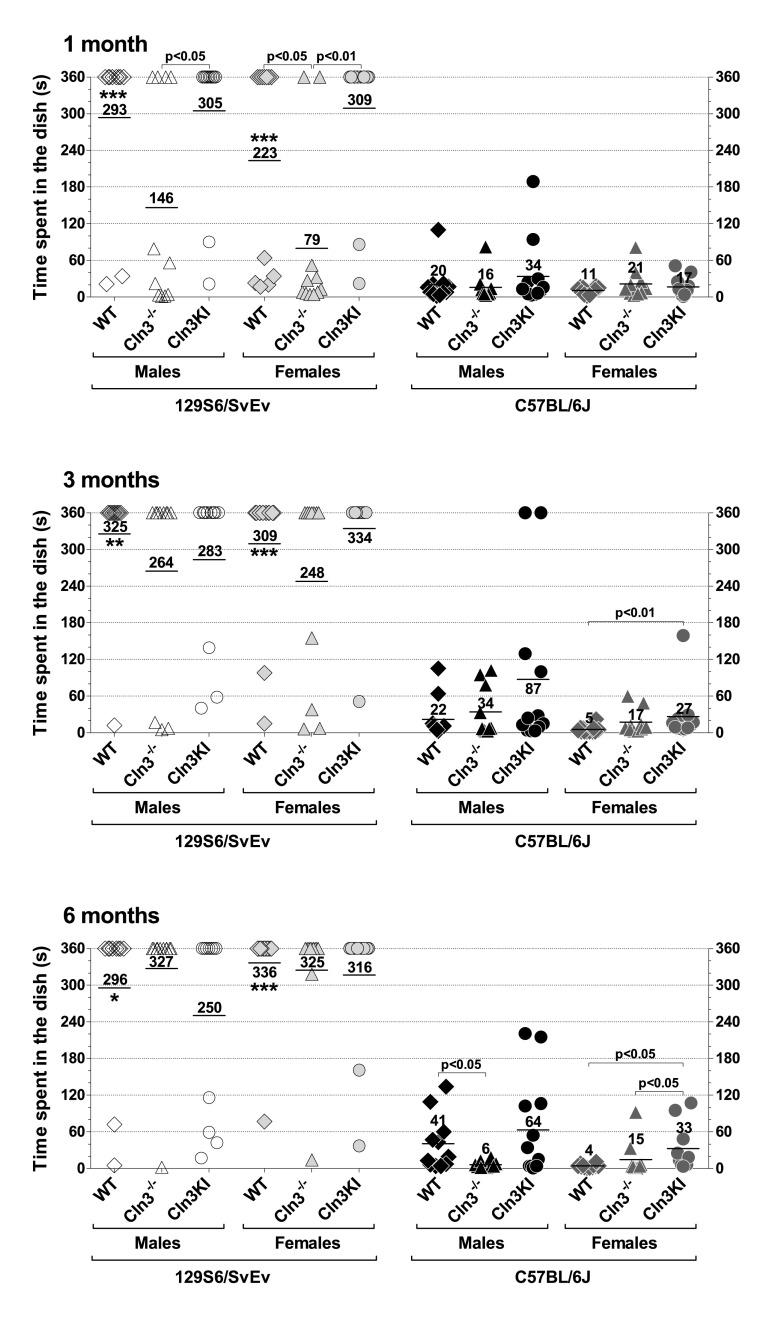
***Cln3*^−/−^ and *Cln3^Δex7/8^*-knock-in mice display genetic-background-, gender- and age-dependent differences in exploratory activity.** The exploratory behavior of 1-, 3- and 6-month-old *Cln3*^−/−^ and *Cln3^Δex7/8^*-knock-in (Cln3KI) mice on two different genetic backgrounds (129S6/SvEv and C57BL/6J) was assessed in the dish test. 129S6/SvEv and C57BL/6J wild-type (WT) mice served as controls. Mice were placed in a large plastic Petri dish (diameter: 15 cm; height: 1.5 cm), and the time until they stepped out of the dish with all four legs was measured. The time limit of the test was 6 minutes. Each symbol represents an individual mouse, and the horizontal bars and numbers show the mean values (*n*=10–12). The same mice were tested at 1, 3 and 6 months of age. Statistical significance was determined by the nonparametric Kruskal-Wallis test with Dunn’s post-test for multiple comparisons. Asterisks indicate significant differences between 129S6/SvEv and C57BL/6J WT mice, males versus males, and females versus females: *P<0.05, **P<0.01 and ***P<0.001.

The exploratory behavior of mice was assessed in the dish test, measuring the time that a mouse stayed in a large Petri dish. It should be noted, however, that activity level and anxiety also affect the outcome of the dish test. In this test, we found a large difference between the two wild-type (WT) strains: whereas most 129S6/SvEv mice, both males and females, stayed in the Petri dish for 6 minutes (the time limit of the test), the majority of C57BL/6J mice left the dish in seconds (Fig. 1, asterisks indicate the statistically significant differences between the two WT strains, comparing males to males, and females to females). Because of this extreme difference, results on the two genetic backgrounds are described separately.

#### Results on the 129S6/SvEv background

One-month-old *Cln3*^−/−^ mice, both males and females, spent considerably less time in a large Petri dish than *Cln3^Δex7/8^* or WT mice (Fig. 1, top graph). At the age of 3 or 6 months, however, the exploratory activity of WT, *Cln3*^−/−^ and *Cln3^Δex7/8^* mice was similar (Fig. 1, middle and bottom graphs).

#### Results on the C57BL/6J background

At both 1 and 3 months of age there were no differences in the exploratory behavior of *Cln3*^−/−^ and *Cln3^Δex7/8^* mice (Fig. 1, top and middle graph). Three-month-old *Cln3^Δex7/8^* females, however, showed decreased exploratory activity as compared to C57BL/6J WT females (Fig. 1, middle graph). Similarly, the exploratory activity of 6-month-old *Cln3^Δex7/8^* females was decreased compared to WT, and this time to *Cln3*^−/−^ females as well (Fig. 1, bottom graph). At the same time, 6-month-old *Cln3*^−/−^ males showed enhanced exploratory activity in comparison to C57BL/6J WT males (Fig. 1, bottom graph). The difference between 6-month-old *Cln3*^−/−^ and *Cln3^Δex7/8^* males did not reach statistical significance (Fig. 1, bottom graph).

### Genetic-background-, gender- and age-dependent differences in the motor phenotypes of *Cln3*^−/−^ and *Cln3^Δex7/8^*-knock-in mice

After the dish test, the same mice were also tested in a modified vertical pole test. This test measures the balance, spatial orientation and motor coordination, although anxiety or motivation to move can also affect the test results.

#### Climbing down the vertical pole

**Fig. 2.**
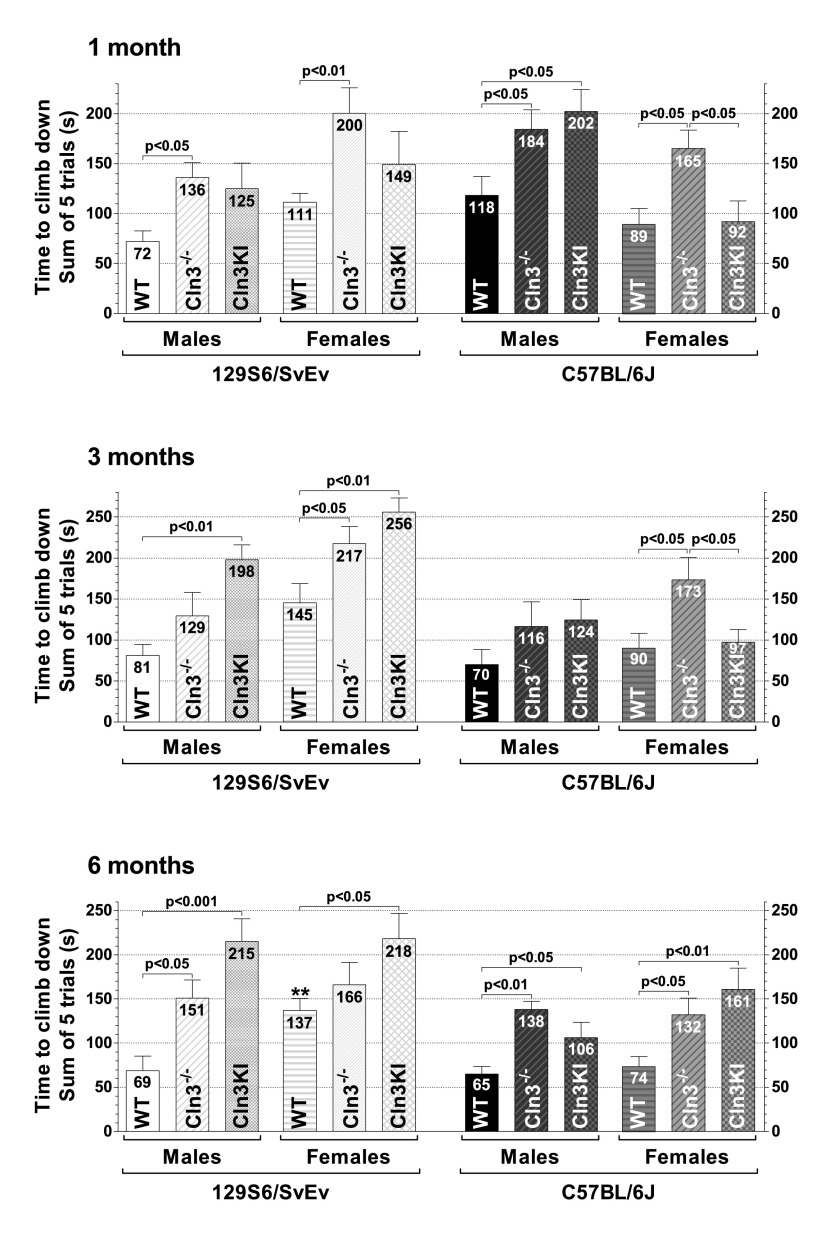
**Genetic-background-, gender- and age-dependent differences in the motor phenotype of *Cln3*^−/−^ and *Cln3^Δex7/8^*-knock-in mice – climbing down a vertical pole.** The ability of the mice to climb down a vertical pole was examined. One-, 3- and 6-month-old *Cln3*^−/−^ and *Cln3^Δex7/8^*-knock-in (Cln3KI) mice on two different genetic backgrounds (129S6/SvEv and C57BL/6J), as well as 129S6/SvEv and C57BL/6J WT mice, were tested. Mice were placed, head downwards, on top of a 59-cm-long all-thread metal pole, and the time until the mice climbed down to the base of the pole was measured in five consecutive trials. Data are plotted as the total time of the five trials. Columns and bars represent mean±s.e.m. (*n*=10–12). The same mice were tested at 1, 3 and 6 months of age. Statistical significance was determined by 1-way ANOVA with Bonferroni’s post-test for multiple comparisons. Asterisks indicate the significant difference between 129S6/SvEv and C57BL/6J WT females at 6 months of age: **P<0.01.

First, the ability of mice to climb down a vertical pole was examined (Fig. 2). Whereas WT mice climbed down the pole quickly and touched the base of the pole without hesitation, *Cln3*^−/−^ and *Cln3^Δex7/8^* mice showed abnormal behavior, which included turning upward, falling off the pole, slowly descending, freezing on the pole and hesitating to touch the base of the pole. *Cln3*^−/−^ males on both genetic backgrounds climbed down significantly slower than their WT counterparts at 1 and 6 months of age. Whereas *Cln3^Δex7/8^* males on the C57BL/6J background showed the same age-dependent motor deficit as *Cln3*^−/−^ males (at 1 and 6 months), *Cln3^Δex7/8^* males on the 129S6/SvEv background exhibited a marked delay in climbing down at 3 and 6 months as compared to WT males (Fig. 2). The differences between *Cln3*^−/−^ and *Cln3^Δex7/8^* males on either genetic background did not reach statistical significance at 1, 3 or 6 months. The climbing-down ability of *Cln3*^−/−^ and *Cln3^Δex7/8^* females, however, varied depending on age and genetic background. On the C57BL/6J background, 1- and 3-month-old *Cln3*^−/−^ females climbed down the pole dramatically slower than *Cln3^Δex7/8^* or WT females (Fig. 2, top and middle graphs). At 6 months, however, *Cln3*^−/−^ and *Cln3^Δex7/8^* females (on C57BL/6J) showed similar motor deficits as compared to WT females (Fig. 2, bottom graph). Although the climbing-down time of *Cln3*^−/−^ and *Cln3^Δex7/8^* females on the 129S6/SvEv background was not statistically different at 1, 3 or 6 months, *Cln3*^−/−^ and *Cln3^Δex7/8^* females showed age-dependent differences in comparison to WT females. *Cln3*^−/−^ (129S6/SvEv) females showed motor deficits at 1 and 3 months, whereas *Cln3^Δex7/8^* (129S6/SvEv) females climbed down significantly slower than 129S6/SvEv WT females at 3 and 6 months (Fig. 2). The two WT strains performed similarly in the climbing-down test, with the only exception that, at 6 months of age, 129S6/SvEv females reached the bases of the pole significantly later than C57BL/6J females (Fig. 2, bottom graph).

#### Turning downward on the vertical pole

**Fig. 3.**
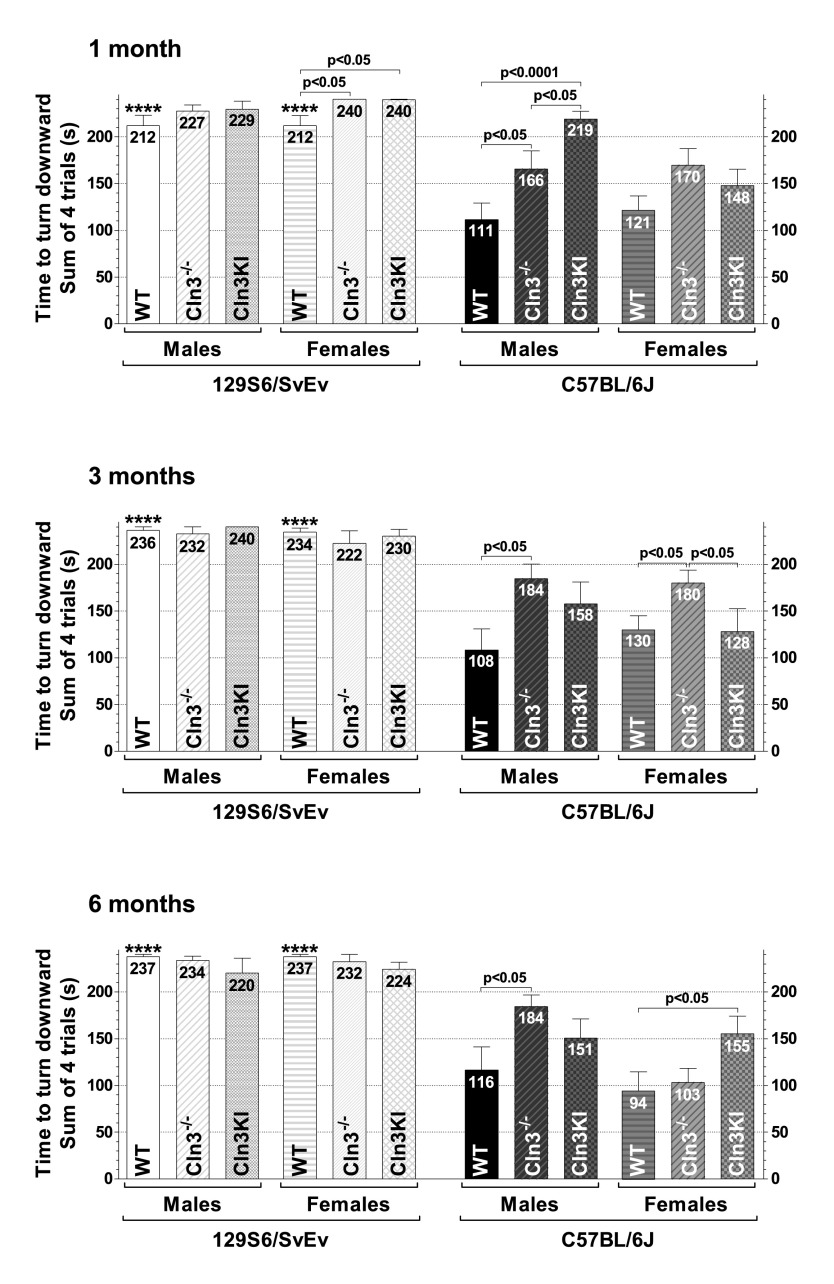
**Genetic-background-, gender- and age-dependent differences in the motor phenotype of *Cln3*^−/−^ and *Cln3^Δex7/8^*-knock-in mice – turning downward on a vertical pole.** The ability of the mice to turn completely downward on a vertical pole was examined. One-, 3- and 6-month-old *Cln3*^−/−^ and *Cln3^Δex7/8^*-knock-in (Cln3KI) mice on two different genetic backgrounds (129S6/SvEv and C57BL/6J), as well as 129S6/SvEv and C57BL/6J WT mice, were tested. Mice were placed, head upward, on top of an all-thread metal pole, and the time until the mice turned completely downward was measured in four consecutive trials. Data are plotted as the total time of the four trials. Columns and bars represent mean±s.e.m. (*n*=10–12). The same mice were tested at 1, 3 and 6 months of age. Statistical significance was determined by 1-way ANOVA with Bonferroni’s post-test for multiple comparisons. Asterisks indicate significant differences between 129S6/SvEv and C57BL/6J WT mice, males versus males, and females versus females: ****P<0.0001.

The ability of mice to turn downward on a vertical pole was also tested. There was a huge difference between 129S6/SvEv and C57BL/6J WT mice: the majority of 129S6/SvEv mice (both males and females) did not turn downward at all during the test trials (Fig. 3). On the 129S6/SvEv background, only 1-month-old *Cln3*^−/−^ and *Cln3^Δex7/8^* females exhibited a phenotype: a statistically significant delay in turning downward as compared to WT females (Fig. 3, top graph).

On the C57BL/6J background, *Cln3*^−/−^ males at all three ages turned downward substantially later than WT males, whereas *Cln3^Δex7/8^* males were only different from WT males at 1 month of age (Fig. 3). The turning-downward performance of 1-month-old *Cln3^Δex7/8^* males, however, was significantly worse than that of 1-month-old *Cln3*^−/−^ males (Fig. 3, top graph). *Cln3*^−/−^ females on the C57BL/6J background had an impaired ability to turn downward at 3 months of age as compared to their WT or *Cln3^Δex7/8^* counterparts (Fig. 3, middle graph). In contrast, at 6 months, *Cln3^Δex7/8^* females (on C57BL/6J) showed a considerable delay in turning downward, whereas *Cln3*^−/−^ females (on C57BL/6J) performed like WTs (Fig. 3, bottom graph).

#### Rotarod test

**Fig. 4.**
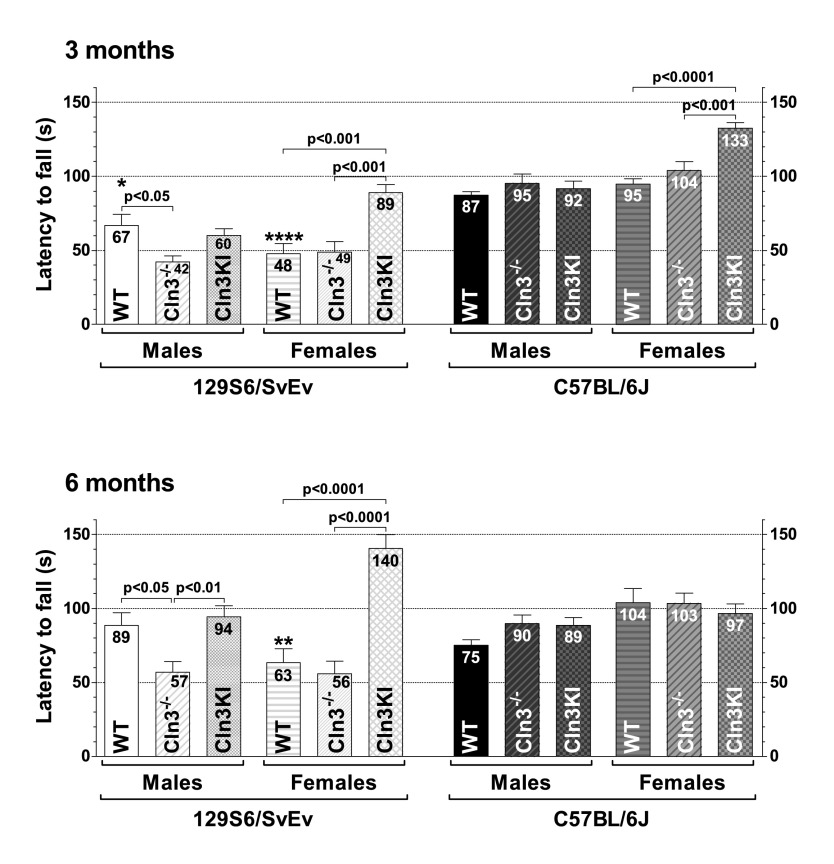
**Only *Cln3***^−/−^ **males on the 129S6/SvEv background show impaired motor coordination in the rotarod test.** Three- and 6-month-old *Cln3*^−/−^ and *Cln3^Δex7/8^*-knock-in (Cln3KI) mice on two different genetic backgrounds (129S6/SvEv and C57BL/6J), as well as 129S6/SvEv and C57BL/6J WT mice, were tested in an accelerating rotarod test (0.2 rpm/s starting from 0 rpm). *Cln3*^−/−^, but not *Cln3^Δex7/8^*-knock-in, males on the 129S6/SvEv background fell from the rotating rod significantly sooner than WT males. Columns and bars represent mean±s.e.m. (*n*=10–12). The same mice were tested at 3 and 6 months of age. Statistical significance was determined by 1-way ANOVA with Bonferroni’s post-test for multiple comparisons. Asterisks indicate significant differences between 129S6/SvEv and C57BL/6J WT mice, males versus males, and females versus females: *P<0.05, **P<0.01 and ****P<0.0001.

The rotarod test assesses balance and motor coordination, although motor learning capability and endurance level can also affect the rotarod performance. The sensitivity of the rotarod test depends on the task parameters, particularly on the acceleration (Carter et al., 1999; Pallier et al., 2009; Rustay et al., 2003). After trying different rotarod protocols and accelerations in a previous study (Kovács and Pearce, 2013), we found that a 0.2 rpm/s acceleration is the most suitable to detect even slight differences in rotarod performance. At 3 and 6 months of age, 1 day after the dish test, vertical pole test and tail suspension test, the same mice were also tested in an accelerating rotarod test (0.2 rpm/s starting from 0 rpm). At both 3 and 6 months, only *Cln3*^−/−^ males on the 129S6/SvEv background showed impaired rotarod performance (Fig. 4). Our findings that 3-month-old *Cln3*^−/−^ (129S6/SvEv) females, 3- and 6-month-old *Cln3^Δex7/8^* (129S6/SvEv) males and females, 3- and 6-month-old *Cln3*^−/−^ (C57BL/6J) males and females, and 6-month-old *Cln3^Δex7/8^* (C57BL/6J) males and females exhibit a motor deficit in the vertical pole test (Figs 2, 3), but not in the rotarod test (Fig. 4), show, in agreement with previous studies (Abramow-Newerly et al., 2006; Berman et al., 2011), that the vertical pole test is more sensitive than the rotarod test to detect motor deficits.

A surprising result of the rotarod test was that 3-month-old *Cln3^Δex7/8^* females on both genetic backgrounds and 6-month-old *Cln3^Δex7/8^* (129S6/SvEv) females could stay on the rotating rod markedly longer than their WT and *Cln3*^−/−^ counterparts (Fig. 4). The same mice in the vertical pole test, however, showed either motor deficits [3- and 6-month-old *Cln3^Δex7/8^* (129S6/SvEv) females, 6-month-old *Cln3^Δex7/8^* (C57BL/6J) females] or were not different from WTs [3-month-old *Cln3^Δex7/8^* (C57BL/6J) females] (see Figs 2, 3). Based on the above, a possible explanation for the extraordinary rotarod performance of *Cln3^Δex7/8^* females is that the *Cln3^Δex7/8^*-derived truncated CLN3 increased the motivation of staying on the rotating rod and/or the endurance specifically in females.

We also compared the rotarod performance of the two WT strains and found that 3-month-old 129S6/SvEv males and females, and 6-month-old 129S6/SvEv females, fell from the rotating rod significantly sooner than their C57BL/6J counterparts (Fig. 4).

### Genetic-background-, gender- and age-specific differences in the depressive-like behavior of *Cln3*^−/−^ and *Cln3^Δex7/8^*-knock-in mice

**Fig. 5.**
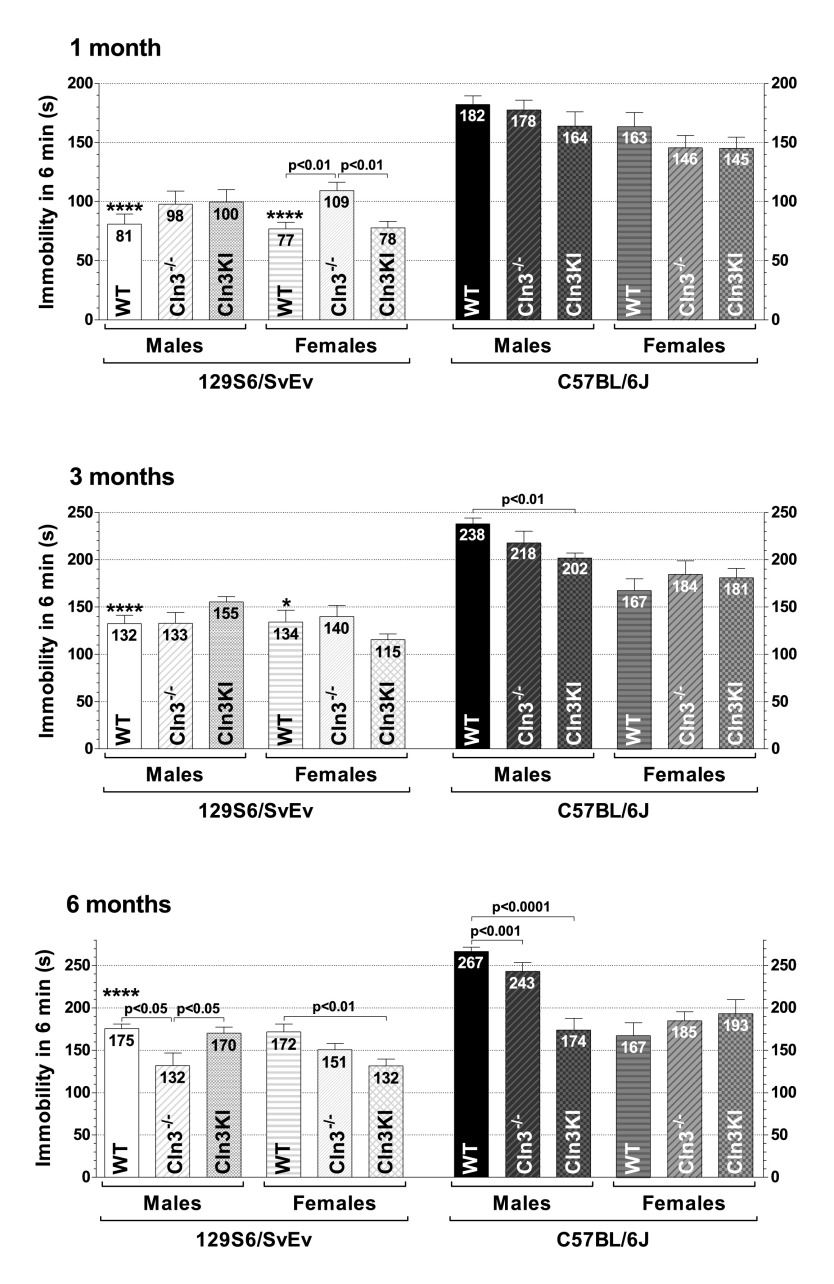
**Genetic-background-, gender- and age-specific differences in the depressive-like behavior of *Cln3*^−/−^ and *Cln3^Δex7/8^*-knock-in mice.** The depressive-like behavior of 1-, 3- and 6-month-old *Cln3*^−/−^ and *Cln3^Δex7/8^*-knock-in (Cln3KI) mice on two different genetic backgrounds (129S6/SvEv and C57BL/6J) was assessed by the tail suspension test. 129S6/SvEv and C57BL/6J WT mice served as controls. Mice were suspended 45 cm above table level, and the duration of immobility was measured in 1-minute bins for 6 minutes. The graphs show the total time of immobility in the 6 minutes of the test; columns and bars represent mean±s.e.m. (*n*=10–12). The same mice were tested at 1, 3 and 6 months of age. Statistical significance was determined by 1-way ANOVA with Bonferroni’s post-test for multiple comparisons. Asterisks indicate significant differences between 129S6/SvEv and C57BL/6J WT mice, males versus males, and females versus females: *P<0.05 and ****P<0.0001.

**Fig. 6.**
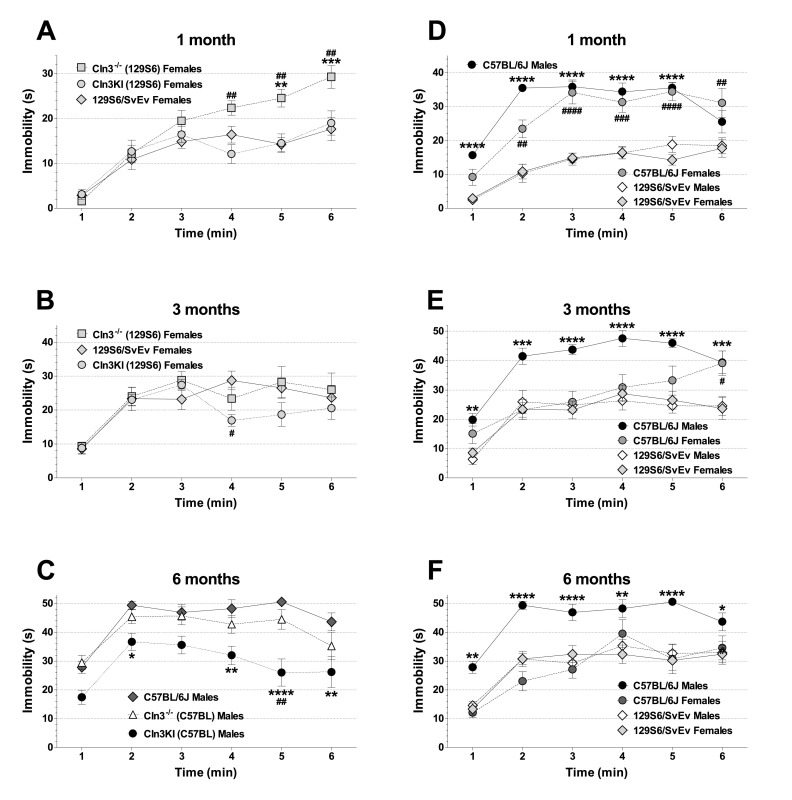
**Differences in the time course of immobility at 1, 3 and 6 months of age.** Mice were suspended by their tail 45 cm above table level, and the duration of immobility was measured in 1-minute bins for 6 minutes. Symbols and bars represent mean±s.e.m. (*n*=10–12). Statistical significance was determined by repeated measures 2-way ANOVA with Bonferroni’s post-test for pairwise multiple comparisons. (A) On the 129S6/SvEv background, 1-month-old *Cln3*^−/−^ females spent more time immobilized than *Cln3^Δex7/8^*-knock-in (Cln3KI) females (^##^*P*<0.01) or WT females (**P<0.01, ***P<0.001) in the 4th, 5th and 6th minutes of the test. (B) On the 129S6/SvEv background, 3-month-old *Cln3*^−/−^ females stayed immobilized significantly longer than *Cln3^Δex7/8^*-knock-in (Cln3KI) females in the 4th minute of the test (^#^*P*<0.05). (C) Six-month-old *Cln3^Δex7/8^*-knock-in (Cln3KI), but not *Cln3*^−/−^, males on the C57BL/6J background spent significantly less time immobilized than C57BL/6J WT males in the 2nd, 4th, 5th and 6th minutes of the test (*P<0.05, **P<0.01, ****P<0.0001). The difference between *Cln3^Δex7/8^*-knock-in (Cln3KI) and *Cln3*^−/−^ males is only statistically significant in the 5th minute (^##^*P*<0.01). (D) One-month-old C57BL/6J WT mice, both males and females, stayed immobilized significantly longer than 129S6/SvEv WT mice. C57BL/6J males versus 129S6/SvEv males: ****P<0.0001; C57BL/6J females versus 129S6/SvEv females: ^##^*P*<0.01, ^###^*P*<0.001, ^####^*P*<0.0001. (E) Three-month-old C57BL/6J WT males stayed immobilized considerably longer than 129S6/SvEv WT males in every minute of the test (**P<0.01, ***P<0.001, ****P<0.0001). The difference between C57BL/6J and 129S6/SvEv WT females is only statistically significant in the 6th minute (^#^*P*<0.05). (F) Six-month-old C57BL/6J WT males stayed immobilized considerably longer than 129S6/SvEv WT males in every minute of the test (*P<0.05, **P<0.01, ****P<0.0001). No difference between 6-month-old C57BL/6J and 129S6/SvEv WT females was found.

The tail suspension test was used to assess the depressive-like behavior of mice. The duration of immobility was measured in 1-minute bins for 6 minutes. Fig. 5 shows the total times of immobility (in 6 minutes). At 1 month of age, *Cln3*^−/−^ (129S6/SvEv) females behaved more depressively, staying immobilized significantly longer than *Cln3^Δex7/8^* (129S6/SvEv) or WT females (Fig. 5, top graph). This difference in total immobility time could not be detected at 3 or 6 months (Fig. 5, middle and bottom graphs). The time course plot revealed significantly increased immobility of *Cln3*^−/−^ (129S6/SvEv) females in the 4th–6th minutes at 1 month (Fig. 6A) and in the 4th minute at 3 months (Fig. 6B).

At 3 months of age, *Cln3^Δex7/8^* (C57BL/6J) males showed abnormally increased desperation and spent markedly less time immobilized than C57BL/6J males (Fig. 5, middle graph).

At 6 months of age, *Cln3*^−/−^ (129S6/SvEv) males spent significantly less time immobilized than *Cln3^Δex7/8^* (129S6/SvEv) or WT males (Fig. 5, bottom graph). Furthermore, 6-month-old *Cln3^Δex7/8^* (129S6/SvEv) females, *Cln3*^−/−^ (C57BL/6J) males and especially *Cln3^Δex7/8^* (C57BL/6J) males exhibited abnormally increased desperation and stayed immobilized for a shorter time than their WT counterparts (Fig. 5, bottom graph; Fig. 6C). The time course plot revealed a statistically significant difference between *Cln3^Δex7/8^* (C57BL/6J) and *Cln3*^−/−^ (C57BL/6J) males in the 5th minute (Fig. 6C).

We also compared the depressive-like behavior of the two WT strains and found that 1- and 3-month-old C57BL/6J males and females, and 6-month-old C57BL/6J males stayed immobilized substantially longer than their 129S6/SvEv counterparts (Fig. 5; Fig. 6D–F).

All the other time courses of immobility not presented in Fig. 6 are shown in supplementary material Figs S1–S3.

### Genetic-background-, gender- and age-specific differences in the weight of *Cln3*^−/−^ and *Cln3^Δex7/8^*-knock-in mice

**Fig. 7.**
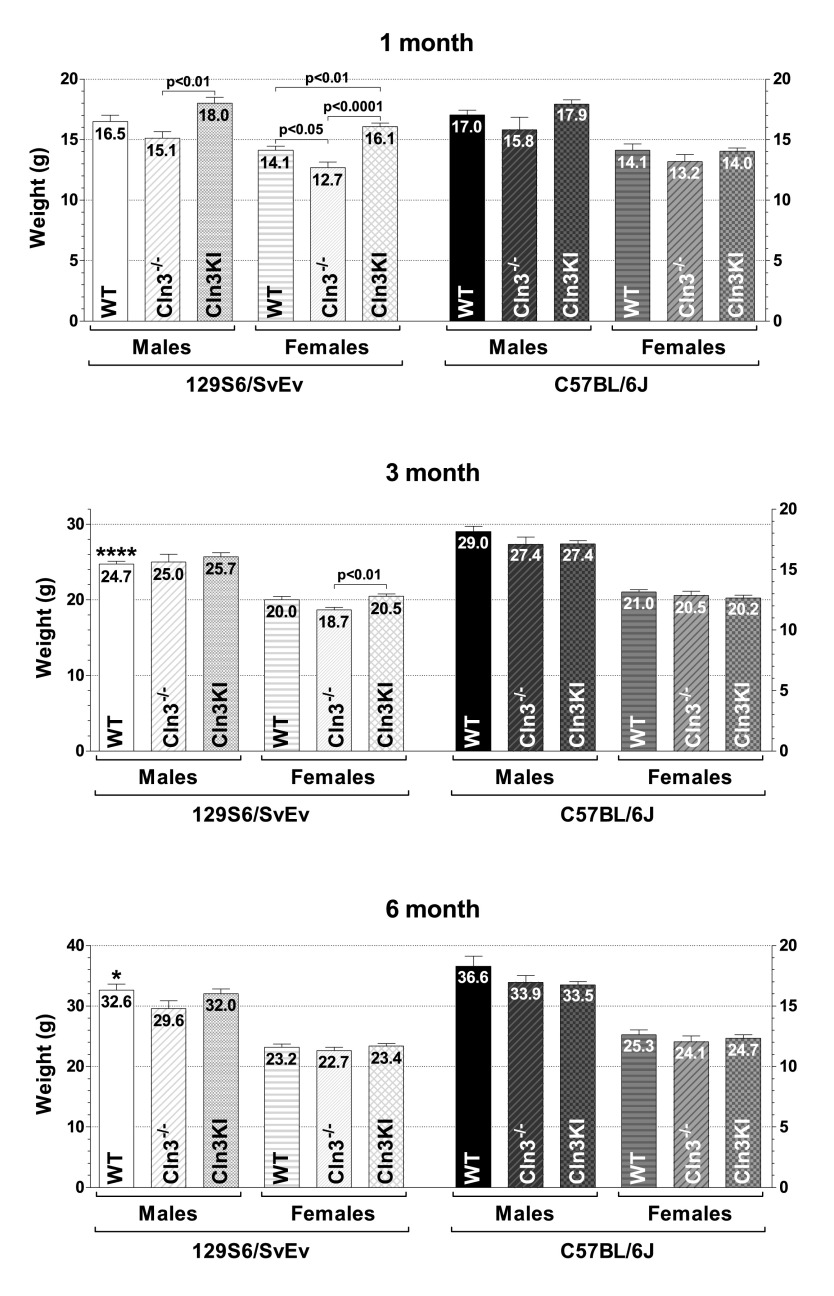
**Genetic-background-, gender- and age-specific differences in the weight of *Cln3*^−/−^ and *Cln3^Δex7/8^*-knock-in (Cln3KI) mice.** The weight of mice used in the behavioral tests was measured. The same mice were weighed at 1, 3 and 6 months of age. Columns and bars represent mean±s.e.m. (*n*=10–12). Statistical significance was determined by 1-way ANOVA with Bonferroni’s post-test for multiple comparisons. Asterisks indicate significant differences between 129S6/SvEv and C57BL/6J WT males: *P<0.05 and ****P<0.0001.

For weight comparison, mice were weighed 30–40 minutes before starting the behavioral tests with them. The same mice were weighed at 1, 3 and 6 months of age. On the C57BL/6J genetic background, no weight differences were found between *Cln3*^−/−^ and *Cln3^Δex7/8^* mice or between WT and mutant mice at any age (Fig. 7). On the 129S6/SvEv background, 1-month-old *Cln3^Δex7/8^* mice, both males and females, were strikingly heavier than *Cln3*^−/−^ mice (Fig. 7, top graph). One-month-old *Cln3^Δex7/8^* (129S6/SvEv) females were also markedly heavier than WT 129S6/SvEv females. At the same time, 1-month-old *Cln3*^−/−^ (129S6/SvEv) females were significantly lighter than 129S6/SvEv females (Fig. 7, top graph). At 3 months of age, *Cln3^Δex7/8^* (129S6/SvEv) females were considerably heavier than *Cln3*^−/−^ (129S6/SvEv) females (Fig. 7, middle graph). Because the observed weight differences might affect the performance in behavioral tests, particularly in motor skill tests, we carried out correlation analyses between the weight and the behavioral test results. The weight of 1- and 3-month-old 129S6/SvEv, *Cln3*^−/−^ (129S6/SvEv) and *Cln3^Δex7/8^* (129S6/SvEv) mice did not correlate with their performance in any of the behavioral tests, indicating that, in our study, the weight differences did not influence the behavioral test results.

We also compared the weights in the two WT strains and found that 129S6/SvEv males were significantly lighter than C57BL/6J males at 3 and 6 months (Fig. 7, middle and bottom graph). Moreover, independently of the genotype and age, males were always substantially heavier than females and the gender-specific weight difference increased with age (supplementary material Fig. S4).

## DISCUSSION

### Mouse models of juvenile CLN3 disease: suitablity for therapeutic studies

In the present study, we compared the exploratory activity, motor function and depressive-like behavior of *Cln3*^−/−^ and *Cln3^Δex7/8^*-knock-in mice on two different genetic backgrounds (129S6/SvEv and C57BL/6J) to determine which mouse model on which genetic background has the most pronounced neurological phenotypes that can be used as outcome measures for therapeutic studies. Based on our results, *Cln3*^−/−^ male mice on the 129S6/SvEv genetic background are the most appropriate candidates for therapeutic studies. They show motor deficits at 1 and 6 months of age in the vertical pole test (Fig. 2), and they were the only mice exhibiting impaired motor coordination in the rotarod test at both 3 and 6 months (Fig. 4). *Cln3*^−/−^ males on the C57BL/6J background and *Cln3^Δex7/8^* males on the 129S6/SvEv background also provide good outcome measures for therapeutic interventions. *Cln3*^−/−^ (C57BL/6J) males had serious difficulties in climbing down (at 1 and 6 months) and turning downward on (at 1, 3 and 6 months) the vertical pole (Figs 2–3), whereas *Cln3^Δex7/8^* (129S6/SvEv) males climbed down the vertical pole drastically slower than WT males at 3 and 6 months of age (Fig. 2). It should be noted that various other behavioral tests (e.g. cognitive tests, open field, light-dark box, gait analysis, variations of rotarod) not used in our study could provide additional valuable readouts for one or more of the models. Similarly, other genetic backgrounds might prove superior in resolving CLN3-deficient behavioral phenotypes.

Our current selection of the most appropriate mouse models was based solely on behavioral tests. Because mouse models of juvenile CLN3 disease show age-dependent neuropathological changes, including microglial activation, astrocytosis and localized neuronal loss (Cotman et al., 2002; Pontikis et al., 2004; Pontikis et al., 2005; Weimer et al., 2009), brain pathology is another important (but terminal) outcome measure for therapeutic studies. Further studies will determine whether the neurological deficits in our chosen models correlate with the severity and progression of neuropathological changes.

### Similarities and differences in the neurological phenotypes of *Cln3*^−/−^ and *Cln3^Δex7/8^*-knock-in mice

The mouse *Cln3^Δex7/8^* gene mimics the most frequent disease-causing human mutation (Cotman et al., 2002). The mutant *Cln3^Δex7/8^* (mouse) and *CLN3^Δex7/8^* (human) genes theoretically produce a truncated protein, which might have a residual function (Kitzmüller et al., 2008). Evidence suggests, however, that cellular quality-control mechanisms at the RNA and protein levels degrade the mutant human and mouse transcript and polypeptide (Chan et al., 2008), and, thus, both *CLN3^Δex7/8^* (human) and *Cln3^Δex7/8^* (mouse) are likely null mutations. If *Cln3^Δex7/8^* is indeed a null mutation then the behavioral phenotypes and their progression in the *Cln3*^−/−^ and *Cln3^Δex7/8^*-knock-in mouse models of juvenile Batten disease should be very similar. Our results show that, although in many cases the behavior of *Cln3*^−/−^ and *Cln3^Δex7/8^* mice was similar, genetic-background-, gender- and age-dependent differences existed between the two mouse models, *Cln3*^−/−^ mice showing more severe phenotypes in some cases whereas *Cln3^Δex7/8^* mice exhibited more pronounced behavioral deficits in other instances. This could suggest that, in *Cln3^Δex7/8^* mice, a truncated *Cln3* transcript has enough expression to impart a residual but presumably partially functional *Cln3* gene product. In a few instances, however, the behavioral defect was more severe or only exhibited in *Cln3^Δex7/8^* mice, indicating a detrimental effect of truncated CLN3 in comparison to the complete lack of CLN3 in *Cln3*^−/−^ mice. The slight differences in the genetic background between our *Cln3*^−/−^ and *Cln3^Δex7/8^* mice (see Materials and Methods) might also contribute to the observed behavioral differences.

Recent studies in the *Cln3*^−/−^ and *Cln3^Δex7/8^* mouse models indicate that juvenile Batten disease has a neurodevelopmental component resulting in early disease phenotypes (at ≤1 month of age) in these mice (Osório et al., 2009; Weimer et al., 2009). Some of these early neurological problems, however, might be reduced or eliminated by compensatory mechanisms during later brain development and maturation [see e.g. *Cln3*^−/−^ (129S6/SvEv) males and females in the dish test (Fig. 1); *Cln3*^−/−^ (129S6/SvEv) females, *Cln3*^−/−^ (C57BL/6J) males and *Cln3^Δex7/8^* (C57BL/6J) males in the climb-down test (Fig. 2); *Cln3*^−/−^ (129S6/SvEv) females, *Cln3^Δex7/8^* (129S6/SvEv) females and *Cln3^Δex7/8^* (C57BL/6J) males in the turn-downward test (Fig. 3); and *Cln3*^−/−^ (129S6/SvEv) females in the tail suspension test (Fig. 5)]. Nevertheless, progressive neurological deficits, depending on the genetic background and the disease model, are also evident [see e.g. *Cln3^Δex7/8^* (129S6/SvEv) males in the climb-down test (Fig. 2); *Cln3*^−/−^ (C57BL/6J) males in the turn-downward test (Fig. 3); *Cln3*^−/−^ (129S6/SvEv) males in the rotarod test (Fig. 4); and *Cln3^Δex7/8^* (C57BL/6J) females in the tail suspension test (Fig. 5)].

Our results also indicate that the vertical pole test is the most representative to detect the motor phenotype of juvenile Batten disease. This was the only test that showed deficits for both *Cln3*^−/−^ and *Cln3^Δex7/8^* models on both genetic backgrounds and for both sexes, at least at one age. Furthermore, because WT C57BL/6J mice (in contrast to 129S6/SvEv mice) effectively turned downward on the vertical pole and both *Cln3*^−/−^ and *Cln3^Δex7/8^* mice on the C57BL/6J background showed impaired ability of turning downward, the standard vertical pole test that takes the sum of turn-downward and climb-down time would reveal a stronger motor phenotype for the C57BL/6J background.

### Choice of the genetic background for mouse models of neurodegenerative and neurological diseases

We observed large differences in the behavior of the 129S6/SvEv and C57BL/6J WT strains (Figs 1, 3–5). Our findings in the rotarod test (better performance of C57BL/6J than 129S6/SvEv mice) and in the tail suspension test (dramatically longer immobility of C57BL/6J than 129S6/SvEv mice) are in agreement with previous reports (Cook et al., 2002; Kelly et al., 1998; Miller et al., 2010). Substantial differences in the locomotor activity, stress reactivity and anxiety-related behaviors of 129S6/SvEv and C57BL/6J mice have also been demonstrated (Cook et al., 2002; Paulus et al., 1999; Van Bogaert et al., 2006). The large behavioral differences between the two WT strains highlight the strong influence that genetic background can have on the disease phenotypes of transgenic mice. In our study, for example, *Cln3^Δex7/8^*-knock-in mice only on the 129S6/SvEv background had motor deficits at 3 months of age (Fig. 2). The genetic background, as several studies have shown, can modify, and even suppress, the effect of a transgene or gene deletion (Bilovocky et al., 2003; Duysen and Lockridge, 2006; Kelly et al., 1998; Lariviere et al., 2001; Lloret et al., 2006; Magara et al., 1999; Mahajan et al., 2004; Nguyen et al., 1997; Tang et al., 2003; Yang et al., 2005).

Although C57BL/6J embryonic stem cell lines became available in recent years, most transgenic mice had been generated using 129S6/SvEv embryonic stem cells. Because 129S6/SvEv mice have a neuroanatomical defect (hypoplasia of the corpus callosum in 60–80% of the mice) (Balogh et al., 1999), carry the Disrupted-in-Schizophrenia 1 (DISC1) mutation (Kim et al., 2012; Koike et al., 2006), and do not perform well in learning tests (Balogh et al., 1999), a common practice has been to backcross 129S6/SvEv transgenic mice to the C57BL/6J genetic background. The C57BL/6J background, however, also has disadvantages. As compared to 129S6/SvEv mice, C57BL/6J mice are hyperactive and aggressive, probably partly due to the fact that the cell surface expression of AMPA (α-amino-3-hydroxy-5-methyl-4-isoxazolepropionate)- and NMDA (N-methyl-D-aspartate)-type glutamate receptors are markedly higher in the brain of C57BL/6J mice (Finn et al., 2010). Furthermore, C57BL/6J mice have a relatively low bone density (Sheng et al., 1999), develop age-related hearing loss (Johnson et al., 1997), and are also susceptible to diet-induced obesity (Collins et al., 2004), type 2 diabetes (Freeman et al., 2006; Parekh et al., 1998) and atherosclerosis (Nishina et al., 1990; Paigen et al., 1985). Because glutamate receptor function is already highly enhanced in C57BL/6J neurons as compared to 129S6/SvEv neurons (Finn et al., 2010), the 129S6/SvEv background seems to be more appropriate for studying neurodegenerative diseases where enhanced glutamate receptor function might be involved in the pathophysiology.

The variable neuroanatomical defect of hypoplastic corpus callosum in 60–80% of 129S6/SvEv mice (Balogh et al., 1999) could have a significant effect in behavioral tests. If this is the case, variation in the test results should be higher on the 129S6/SvEv background (intact corpus callosum in 20–40% of the mice) than on the C57BL/6J background (intact corpus callosum in all mice). In our vertical pole test, rotarod test and tail suspension test, however, as the error bars in Figs 2–5 show, the test result variations on the two genetic backgrounds were very similar.

In summary, our study demonstrates the importance of testing mouse models on different genetic backgrounds and comparing males and females in order to find the most appropriate disease model for therapeutic studies. In addition, we provide the first behavioral comparison of *Cln3*^−/−^ and *Cln3^Δex7/8^*-knock-in mice on identical genetic backgrounds, revealing that the *Cln3^Δex7/8^* mice might differ from *Cln3*^−/−^ mice due to residually expressed truncated CLN3.

## MATERIALS AND METHODS

### Animals

We maintained *Cln3*^−/−^ mice on the 129S6/SvEv genetic background (*Cln3*^−/−^ mice were originally backcrossed with 129S6/SvEv mice for 12 generations), and *Cln3^Δex7/8^*-knock-in mice on the C57BL/6J genetic background (*Cln3^Δex7/8^*-knock-in mice were originally backcrossed with C57BL/6J mice for 12 generations). To compare the two mouse models we backcrossed *Cln3*^−/−^ (129S6/SvEv) mice to the C57BL/6J, and *Cln3^Δex7/8^* (C57BL/6J) mice to the 129S6/SvEv genetic background for ten generations. To verify the genetic background of the mouse strains used in our study, tail snips of representative mice (two males and two females from each strain) were sent to The Jackson Laboratory (Bar Harbor, Maine) for genome scanning. Analysis of 158 SNPs polymorphic between the C57BL/6 and 129 strains and covering 19 autosomes and the X chromosome with a density of ~15–20 Mb showed that our *Cln3*^−/−^ (129S6/SvEv) and *Cln3^Δex7/8^* (129S6/SvEv) mice have 99.4% and 97.6% of their genome, respectively, from the 129S6/SvEv strain, and our *Cln3*^−/−^ (C57BL/6J) and *Cln3^Δex7/8^* (C57BL/6J) mice have 99.4% and 98.1% of their genome, respectively, from the C57BL/6J strain (supplementary material Table S1).

129S6/SvEv and C57BL/6J WT mice maintained in our mouse colony were the controls in the behavioral experiments. Mice were housed in individually vented microisolator cages (four or five mice/cage) with *ad libitum* access to food and water. Mice were fed with the Teklad Global 2918 diet (Harlan Laboratories, Indianapolis, IN), and their drinking water was tap water. All procedures were carried out according to the guidelines of the Animal Welfare Act and NIH policies, and were approved by the Sanford Research Animal Care and Use Committee.

### Behavioral testing

Mice were transported to the behavioral testing room where the lights had been dimmed. Mice were labeled on their tails for easy identification, weighed, and were allowed to acclimatize to the room for at least 20 minutes before starting the behavioral tests. All mice were tested first in the dish test, then in the modified vertical pole test, and finally in the tail suspension test. One day later (in the case of 3- and 6-month-old mice) the same mice were also tested in the rotarod test. All behavioral tests were carried out under dim light to keep the anxiety level of mice minimal. The same mice were tested at 1, 3 and 6 months of age.

#### Dish test

The dish test measures the exploratory behavior of mice, although activity level and anxiety also affect the outcome of this test. The mouse was placed in a large plastic Petri dish (diameter: 15 cm; height: 1.5 cm) located in the middle of a clean table, and the time until the mouse stepped out of the dish with all four legs was measured in a single trial. The time limit of the trial was 6 minutes. The Petri dish was cleaned between mice from the same cage (same genotype and gender), and a new Petri dish was used for each cage.

#### Modified vertical pole test

With the vertical pole, the balance, spatial orientation and motor coordination of mice are tested. However, anxiety or motivation to move might also affect the test results. In the traditional vertical pole test (see e.g. Iwamoto et al., 2003), the mouse is placed on top of the pole head upward, and the time until it turns downwards and climbs down to the base of the pole is measured. Because the majority of our mice on the 129S6/SvEv genetic background, including WT mice, do not turn downward at all (see Fig. 3), we modified the vertical pole test so it starts with the climb down. The vertical pole was an all-thread metal rod (diameter: 1.27 cm; height: 66 cm), screwed to a 3.81-cm-thick plastic block (24.5 cm×25.4 cm). The plastic block was covered with a 3.81-cm-thick green hunting seat cushion (nitrile rubber/PVC foam) to prevent the mice from injury if they fell from the pole. The height of the pole measured from the surface of the hunting seat cushion was 59 cm. The mouse was placed, head downward, on top of the pole, and the time until the mouse climbed down to the base of the pole was measured in five consecutive trials. After the 5th trial, the same mouse was placed, head upward, on top of the pole, and the time until the mouse turned completely downward was measured in four consecutive trials. Each climbing-down and turning-downward trial was terminated after 60 seconds to avoid exhaustion. If the mouse fell from the pole, a trial result of 60 seconds was given. The time to climb down (sum of the five trials in seconds), and the time to turn downward (sum of the four trials in seconds) were calculated for each mouse.

#### Tail suspension test

This test measures the depressive-like behavior of mice (Taniguchi et al., 2009). Rodents subjected to the short-term, inescapable stress of being suspended by their tail develop an immobile posture. Various antidepressant drugs reverse the immobility and promote the occurrence of escape-related behavior (Cryan et al., 2005). Mice were suspended 45 cm above table level by taping their tails to Tygon tubing tightened to an extension support ring on an iron support stand. The duration of immobility (defined as the absence of all movement except for those required for respiration) was measured in 1-minute bins for 6 minutes.

#### Rotarod test

The rotarod measures the ability of the mouse to maintain balance on a motor-driven, rotating rod. Thus, the fore- and hind-limb motor coordination and balance can be analyzed. Motor learning capability and endurance level might also affect the rotarod performance. The rotarod test using two Rotamex-5 accelerating rotarod instruments (Columbus Instruments, Columbus, OH; diameter of the rotating rod: 3 cm) was performed as described previously (Kovács and Pearce, 2013), with some modifications. The start speed of the rotarod was 0 rpm and the acceleration was set to 0.2 rpm/s. The cut-off time was set at 240 seconds but all mice fell from the rotarod way before the set cut-off time. Mice were trained on the rotarod in three consecutive runs. Following training, mice rested for 1.5 hours and then were tested on the rotarod in three test trials each consisting of three consecutive runs, with 15 minutes of rest between the trials. The average latency to fall from the rotating rod in the test trials (average of the nine runs in the three trials) was calculated for each mouse.

### Statistical analysis

Statistical analysis was performed using GraphPad Prism 5.04 (GraphPad Software, San Diego, CA). Most of the data sets from the vertical pole test, rotarod test, tail suspension test and weight measurement passed the normality test (alpha level 0.05); therefore, 1-way ANOVA with Bonferroni’s post-test was used for comparison in these behavioral tests and for weight comparison as well. Because most data sets from the dish test did not pass the normality test (alpha level 0.05), the non-parametric Kruskal-Wallis test with Dunn’s post-test was applied for comparison in this case. To compare the time courses in the tail suspension test, repeated measures 2-way ANOVA with Bonferroni’s post-test was used. Alpha level was 0.05 in all statistical tests.

## Supplementary Material

Supplementary Material
