# Supplementary material for: Finding the most appropriate mouse model of juvenile CLN3 (Batten) disease for therapeutic studies: the importance of genetic background and gender
Source: Dis Model Mech. 2015 Apr;8(4):351–61. doi: 10.1242/dmm.018804 (PMC4381334; doi:10.1242/dmm.018804)
Supplement: Supplementary Material [file supp_8_4_351__index.html]

Finding the most appropriate mouse model of juvenile CLN3 (Batten) disease for therapeutic studies: the importance of genetic background and gender — Supplementary Material 

# Finding the most appropriate mouse model of juvenile CLN3 (Batten) disease for therapeutic studies: the importance of genetic background and gender

## DMM018804 Supplementary Material

**Files in this Data Supplement:**

- **Supplementary Material**
