# Supplementary material for: Finding the most appropriate mouse model of juvenile CLN3 (Batten) disease for therapeutic studies: the importance of genetic background and gender
Source: Dis Model Mech. 2015 Apr;8(4):351–61. doi: 10.1242/dmm.018804 (PMC4381334; doi:10.1242/dmm.018804)
Supplement: Supplementary Material [file supp_8.4.351_DMM018804.pdf]

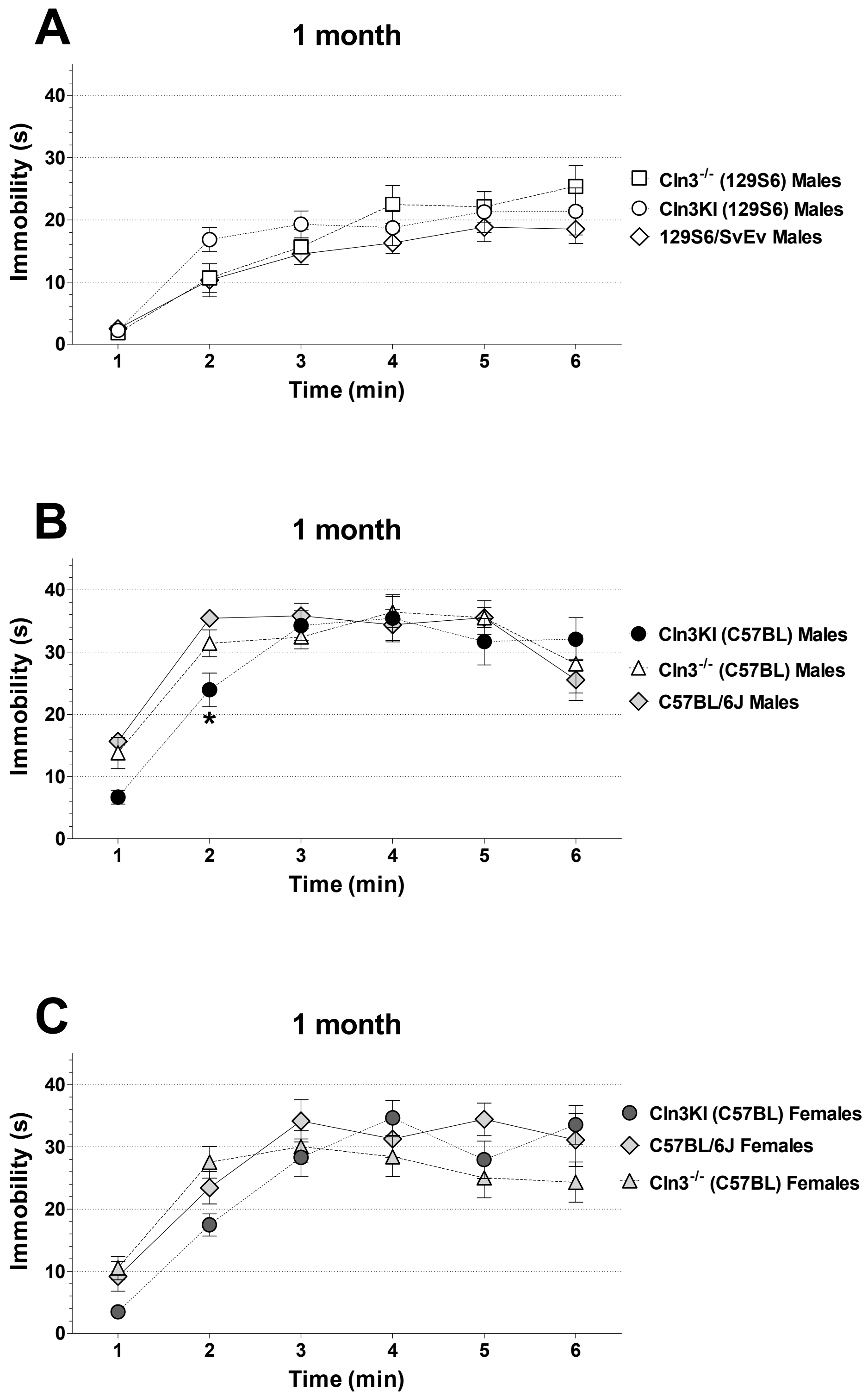

Supplementary Fig. 1. Similarities in the time course of immobility at 1 month of age. One-month-old mice were suspended by their tail 45 cm above table level, and the duration of immobility was measured in 1 minute bins for 6 minutes. Symbols and bars represent mean ± SEM (n=10-12).

Statistical significance was analyzed by repeated measures 2-way ANOVA with Bonferroni's post-test for pairwise multiple comparisons. (**A**) *Cln3*$^{-/-}$ and *Cln3*$^{\Delta ex7/8}$-knock-in (Cln3KI) males on the 129S6/SvEv background, and 129S6/SvEv WT males had very similar time course of immobility. (**B**) *Cln3*$^{-/-}$ and *Cln3*$^{\Delta ex7/8}$-knock-in (Cln3KI) males on the C57BL/6J background, and C57BL/6J WT males had similar time course of immobility. The only difference between *Cln3*$^{\Delta ex7/8}$-knock-in (Cln3KI) and C57BL/6J WT males was in the 2nd minute of the test (*$p<0.05$). (**C**) *Cln3*$^{-/-}$ and *Cln3*$^{\Delta ex7/8}$-knock-in (Cln3KI) females on the C57BL/6J background, and C57BL/6J WT females had similar time course of immobility.

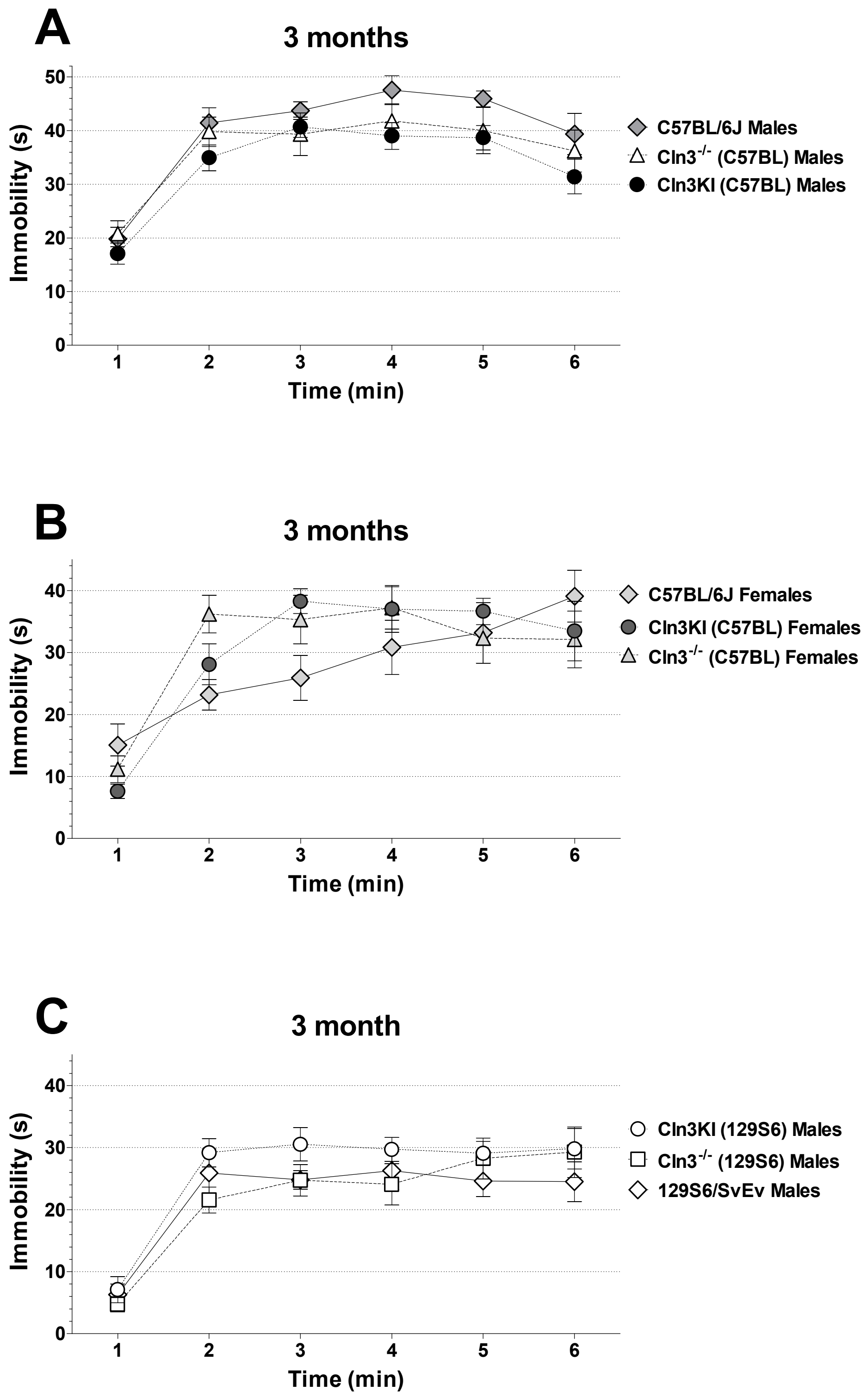

Supplementary Fig. 2. Similarities in the time course of immobility at 3 months of age. Three-month-old mice were suspended by their tail 45 cm above table level, and the duration of immobility was measured in 1 minute bins for 6 minutes. Symbols and bars represent mean ± SEM (n=10-12).

Statistical significance was analyzed by repeated measures 2-way ANOVA with Bonferroni's post-test for pairwise multiple comparisons. (**A**) *Cln3*$^{-/-}$ and *Cln3*$^{\Delta ex7/8}$-knock-in (Cln3KI) males on the C57BL/6J background, and C57BL/6J WT males had very similar time course of immobility (**B**) *Cln3*$^{-/-}$ and *Cln3*$^{\Delta ex7/8}$-knock-in (Cln3KI) females on the C57BL/6J background, and C57BL/6J WT females had similar time course of immobility. (**C**) *Cln3*$^{-/-}$ and *Cln3*$^{\Delta ex7/8}$-knock-in (Cln3KI) males on the 129S6/SvEv background, and 129S6/SvEv WT males had very similar time course of immobility.

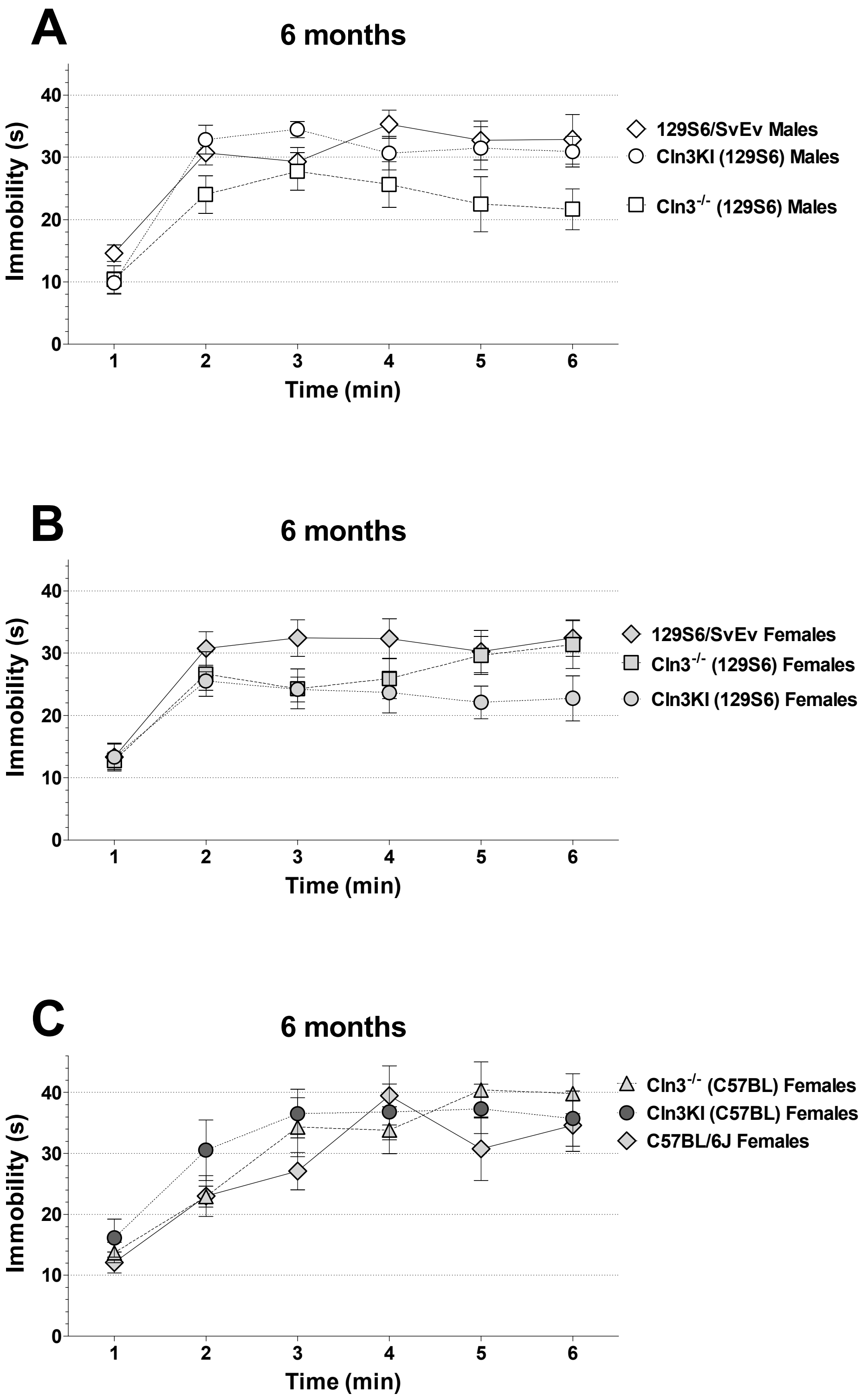

Supplementary Fig. 3. Similarities in the time course of immobility at 6 months of age. Six-month-old mice were suspended by their tail 45 cm above table level, and the duration of immobility was measured in 1 minute bins for 6 minutes. Symbols and bars represent mean ± SEM (n=10-12).

Statistical significance was analyzed by repeated measures 2-way ANOVA with Bonferroni's post-test for pairwise multiple comparisons. (**A**) *Cln3*$^{-/-}$ and *Cln3*$^{\Delta ex7/8}$-knock-in (Cln3KI) males on the 129S6/SvEv background, and 129S6/SvEv WT males had similar time course of immobility. (**B**) *Cln3*$^{-/-}$ and *Cln3*$^{\Delta ex7/8}$-knock-in (Cln3KI) females on the 129S6/SvEv background, and 129S6/SvEv WT females had similar time course of immobility. (**C**) *Cln3*$^{-/-}$ and *Cln3*$^{\Delta ex7/8}$-knock-in (Cln3KI) females on the C57BL/6J background, and C57BL/6J WT females had very similar time course of immobility.

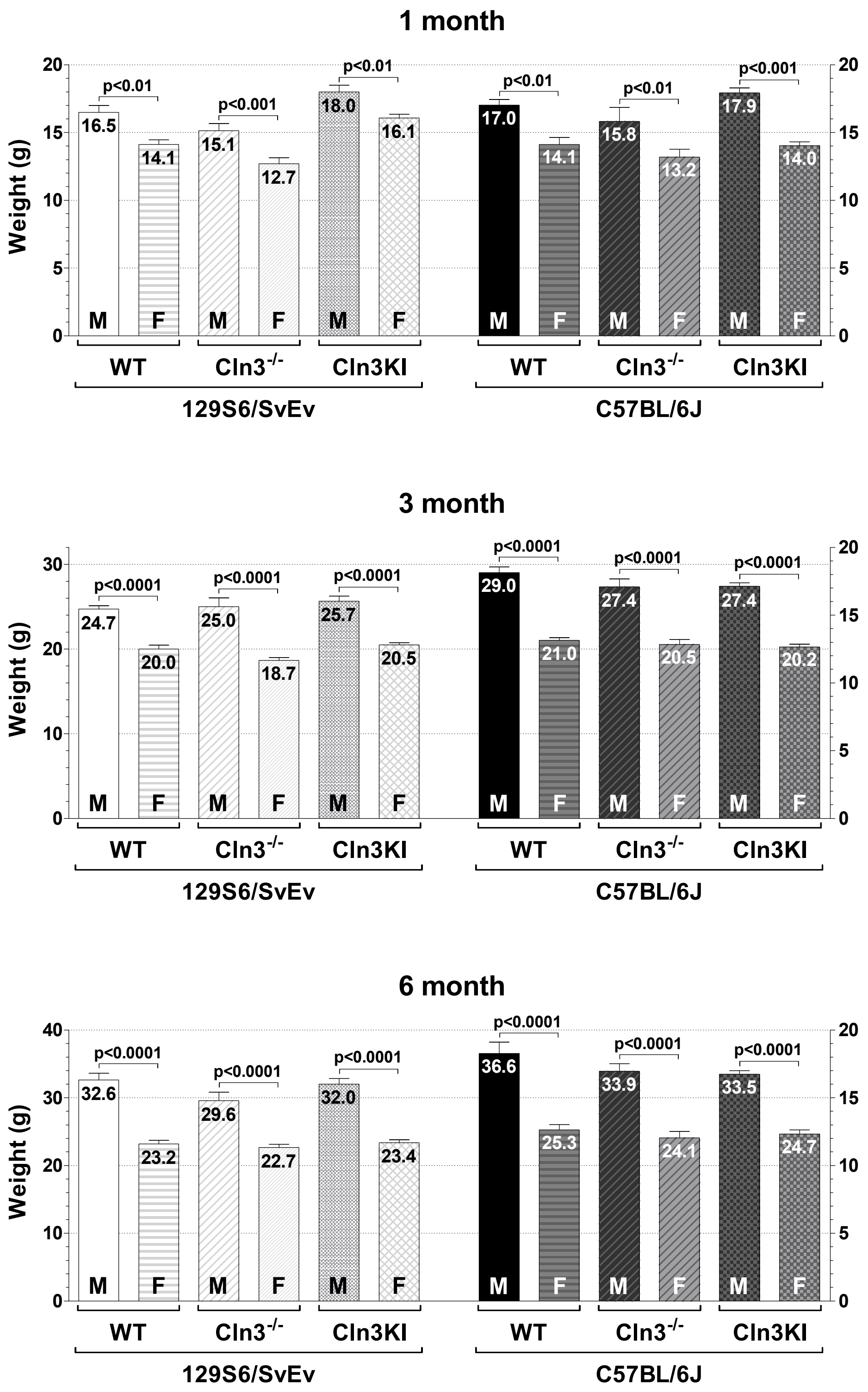

Supplementary Fig. 4. Males are always heavier than females independently of the genotype, genetic background and age. The weight of male (M) and female (F) mice used in the behavioral tests was measured. The same mice were weighed at 1, 3 and 6 months of age. Columns and bars

represent mean ± SEM (n=10-12). Statistical significance was determined by 1-way ANOVA with Bonferroni's post-test for multiple comparisons.

Supplementary Table 1. Genome scanning analysis of representative mice.

		% C57BL/6	0.6%	0.6%	0.6%	0.6%	2.5%	2.5%	2.5%	2.2%	99.4%	99.4%	99.4%	99.4%	98.1%	98.1%	98.1%	98.1%
SNP_ID		% 129	99.4%	99.4%	99.4%	99.4%	97.5%	97.5%	97.5%	97.8%	0.6%	0.6%	0.6%	0.6%	1.9%	1.9%	1.9%	1.9%
			$Cln3^{-/-}$ on 129/SvEv				$Cln3^{\Delta ex7/8}$ on 129/SvEv				$Cln3^{-/-}$ on C57BL/6J				$Cln3^{\Delta ex7/8}$ on C57BL/6J			
Chromosome-bp	C57BL/6	129	female 1	female 2	male 1	male 2	male 1	male 2	female 1	female 2	male 1	male 2	female 1	female 2	male 1	male 2	female 1	female 2
01-005230167-M	C	T	T		T	T	T	T	T	T	C	C	C	C	C	C	C	C
01-026072256-M	G	T	T	T	T	T	T	T/G	T/G	T	G	G	G	G	G	G	G	G
01-046003967-M	A	C	C	C	C	C	C	C	C	C	A	A	A	A	A	A	A	A
01-065042402-M	G	A	A	A	A	A	A	A	A	A	G	G	G	G	G	G	G	G
01-086182722-M	A	G	G	G	G	G	G	G	G	G	A	A	A	A	G	G/A	G/A	G
01-102073421-M	G	C	C	C	C	C	C	C	C	C	G	G	G	G	G	C/G	C/G	G
01-122978406-M	G	T	T	T	T	T	T	T	T	T	G	G	G	G	G	G	G	G
01-143006008-M	A	G	G	G	G	G	G	G	G	G	A	A	A	A	A	A	A	A
01-162977516-M	G	A	A	A	A	A	A	A	A	A	G	G	G	G	G	G	G	G
01-178701443-M	A	G	G	G	G	G	G	G	G	G	A	A	A	A	A	A	A	A
01-193173300-M	G	A	A	A	A	A	A	A	A	A	G	G	G	G	G	G	G	G
02-003179310-M	G	A	A	A	A	A	A	A	A	A	G	G	G	G	G	G	G	G
02-020551513-M	A	G	G	G	G	G	A	A	A	G/A	A	A	A	A	A	A	A	A
02-041042651-M	C	G	G	G	G	G	G	G	G	G	C	C	C	C	C	C	C	C
02-061161692-M	T	C	C	C	C	C	C	C	C	C	T	T	T	T	T	T	T	T
02-078062303-M	T	G	G	G	G	G	G	G	G	G	T	T	T	T	T	T	T	T
02-101074874-M	A	G	G	G	G	G	G	G	G	G	A	A	A	A	A	A	A	
02-119931810-M	A	T	T	T	T	T	T	T	T	T	A	A	A	A	A	A	A	A
02-139603599-M	T	A	A	A	A	A	A	A	A	A	T	T	T	T	T		T	T
02-161858323-M	C	A	A	A	A	A	A	A	A	A	C	C	C	C	C	C		C
02-179086475-M	G	T	T	T	T	T	T	T	T	T	G	G	G	G	G	G	G	G
03-011350737-M	G	C	C	C	C	C	C	C	C	C	G	G	G	G	G		G	G
03-032431792-M	G	A	A	A	A	A	A	A		A	G	G	G	G	G	G	G	G
03-053161686-M	T	C	C	C	C	C	C	C	C	C	T	T	T	T	T	T	T	T
03-073214161-M	A	T	T	T	T	T	T	T	T	T	A	A	A	A	A	A	A	A
03-088155864-N	T	C	C	C	C	C	C	C	C	C	T	T	T	T	T	T	T	T
03-110268748-M	C	T	T	T	T	T	T	T	T	T	C	C	C	C	C	C	C	C
03-131286062-N	C	T	T	T	T	T	T	T	T	T	C	C	C	C	C	C	C	C
03-151125653-M	A	T	T	T	T	T	T	T	T	T	A	A	A	A	A	A	A	A
03-160287200-M	G	A	A	A	A	A	A	A	A	A	G	G	G	G	G	G	G	G
04-003163167-M	G	T	T	T	T	T	T	T	T	T	G	G	G	G	G	G	G	G
04-022875524-M	C	T	T	T	T	T	T	T	T	T	C	C	C	C	C	C	C	C
04-039199957-M	C	G	G	G	G	G	G	G	G	G	C	C	C	C	C	C	C	C
04-061409675-M	G	T	T	T	T	T	T	T	T	T	G	G	G	G	G	G	G	G
04-084241978-M	T	C	C	C	C	C	C	C	C	C	T	T	T	T	T	T	T	T
04-106314110-M	C	A	A	A	A	A	A	A	A	A	C	C	C	C	C	C	C	C
04-120122875-M	G	A	A	A	A	A	A	A	A	A	G	G	G	G	G	G	G	G
04-141084977-M	A	G	G	G	G	G	G	G	G	G	A	A	A	A	A	A	A	A
04-151168886-M	G	T	T	T	T	T	T	T	T	T	G	G	G	G	G	G	G	G
05-003188964-M	C	T	T	T	T	T	T	T	T	T	C	C	C	C	C	C	C	C
05-023890459-M	C	G	G	G	G	G	G	G	G	G	C	C	C	C	C	C	C	C
05-044010971-M	T	G	G	G	G	G	T	G/T	G/T	T	T	T	T	T	T	T	T	T
05-065734378-N	A	G	G	G	G	G	G	G	G	G	A	A	A	A	A	A	A	A
05-082050497-M	C	A	A	A	A	A	A	A	A	A	C	C	C	C	C	C	C	C
05-106058057-M	T	C	C	C	C	C	C	C	C	C	T	T	T	T	T	T	T	T
05-125054661-M	T	C	C	C	C	C	C	C	C	C	T	T	T	T	T	T	T	T
05-139966164-M	T	C	C	C	C	C	C	C	C	C	T	T	T	T	T	T	T	T

05-149044358-M	C	A	A	A	A	A	A	A	A	A	C	C	C	C	C	C	C	C
06-003167392-M	C	T	T	T	T	T	T	T	T	T	C	C	C	C	C	C	C	C
06-017592278-N	G	T	T	T	T	T	T	T	T	T	G	G	G	G	G		G	G
06-045126062-M	A	G	G	G	G	G	G	G	G	G	A	A	A	A	A	A	A	A
06-062599520-M	A	G	G	G	G	G	G	G	G	G	A	A	A	A	A	A	A	A
06-083216741-M	G	T	T	T	T	T	T	T	T	T	G	G	G	G	G	G	G	G
06-106057600-M	C	T	T	T	T	T	T	T	T	T	C	C	C	C	C	C	C	C
06-122941044-M	T	A	A	A	A	A	A	A	A	A	T	T	T	T	T	T	T	T
06-135955068-M	C	A	A	A	A	A	A	A	A	A	C	C	C	C	C	C	C	C
06-149052281-M	C	G	G	G	G	G	G	G	G	G	C	C	C	C	C	C	C	C
07-004201219-N	A	G	G	G	G	G	G	G	G	G	A	A	A	A	A	A	A	A
07-022997618-M	C	T	C	C	C	C	C	C	C	C	C	C	C	C	C	C	C	C
07-044964980-M	G	C	C	C	C	C	C	C	C	C	G	G	G	G	G	G	G	G
07-064095712-M	G	T	T		T	T	T	T	T	T	G	G	G	G	G	G	G	G
07-085095955-M	C	T	T		T	T	T	T	T	T	C	C	C	C	C	C	C	C
07-106153798-M	G	T	T	T	T	T	T	T	T	T	T	T	T	T	T	T	T	T
07-135024189-M	C	T	T	T	T	T	C	C	C	C	C	C	C	C	C	C	C	C
08-003089774-M	T	C	C	C	C	C	C	C	C	C	T	T	T	T	T	T	T	T
08-025034424-M	C	T	T	T	T	T	T	T	T	T	C	C	C	C	C	C	C	C
08-045080531-M	T	C	C	C	C	C	C	C	C	C	T	T	T	T	T	T	T	T
08-067470102-N	G	C	C	C	C	C	C	C	C	C	G	G	G	G	G	G	G	G
08-086985036-M	C	T	T	T	T	T	T	T	T	T	C	C	C	C	C	C	C	C
08-107088585-M	A	G	G	G	G	G	G	G	G	G	A	A	A	A	A	A	A	A
08-123021323-M	T	C	C	C	C	C	C	C	C	C	T	T	T	T	T	T	T	T
09-003938578-M	T	C	C	C	C	C	C	C	C	C	T	T	T	T	T	T	T	T
09-016198772-M	G	T	T	T	T	T	T	T	T	T	G	G	G	G	G	G	G	G
09-034961903-M	G	A	A	A	A	A	A	A	A	A	G	G	G	G	G	G	G	G
09-052894456-M	C	A	A	A	A	A	A	A	A	A	C	C	C	C	C	C	C	C
09-077792491-M	T	A	A	A	A	A	A	A	A	A	T	T	T	T	T	T	T	T
09-098865563-M	T	C	C	C	C	C	C	C	C	C	T	T	T	T	T	T	T	T
09-115037423-M	G	A	A	A	A	A	A	A	A	A	G	G	G	G	G	G	G	G
09-125082704-M	G	A	A	A	A	A	A	A	A	A	G	G	G	G	G	G	G	G
10-003233934-M	G	C	C	C	C	C	C	C	C		G	G	G	G	G	G	G	G
10-023127952-M	A	G	G	G	G	G	G	G	G	G	A	A	A	A	A	A	A	A
10-034328321-G	C	A	A	A	A	A	A	A	A	A	C	C	C	C	C	C	C	C
10-053898997-M	G	C	C	C	C	C	C	C	C	C	G	G		G	G	G	G	G
10-072471838-G	A	G	G	G	G	G	G	G	G	G	A	A	A	A	A	A	A	A
10-093476458-N	T	C	C	C	C	C	C	C	C	C	T	T	T	T	T	T	T	T
10-117629082-M	A	G	G	G	G	G	G	G	G	G	A	A	A	A	A	A	A	A
10-129117100-M	G	A	A	A	A	A	A	A	A	A	G	G	G	G	G	G	G	G
11-005661856-N	T	A	A	A	A	A	A	A	A	A	T	T	T	T	T		T	T
11-024515722-M	G	A	A	A	A	A	A	A	A	A	G	G	G	G	G	G	G	G
11-047224208-M	C	A	A	A	A	A	A	A	A	A	C	C	C	C	C	C	C	C
11-070331570-M	T	C	C	C	C	C	C	C	C	C	T	T	T	T	T	T	T	T
11-090199967-M	T	C	C	C	C	C	C	C	C	C	T	T	T	T	T	T	T	T
11-111226923-M	A	C	C	C	C	C	C	C	C	C	A	A	A	A	A	A	A	A
11-122195777-M	C	T	T	T	T	T	T	T	T	T	C	C	C	C	C	C	C	C
12-003567042-M	G	T	T	T	T	T	T	T	T	T	G	G	G	G	G	G	G	G
12-022232159-M	C	G	G	G	G	G	G	G	G	G	C	C	C	C	C	C	C	C
12-041270227-M	A	G	G	G	G	G	G	G	G	G	A	A	A	A	A	A	A	A
12-061271925-M	A	T	T	T	T	T	T	T	T	T	A	A	A	A	A	A	A	A

12-069389027-M	A	G	G	G	G	G	G	G	G	G	A	A	A	A	A	A	A	A
12-092440423-M	G	C	C	C	C	C	C	C	C	C	G	G	G	G	G	G	G	G
12-107536049-N	A	G	G	G	G	G	G	G	G	G	A	A	A	A	A	A	A	A
12-112276179-M	A	T	T	T	T	T	T	T	T	T	A	A	A	A	A	A	A	A
13-003966099-M	A	G	G	G	G	G	G	G	G	G	A	A		A	A	A	A	A
13-023105764-M	A	G	G	G	G	G	G	G	G	G	A	A	A	A	A	A	A	A
13-043995023-N	T	G	G	G	G	G	G	G	G	G	T	T	T	T	T		T	T
13-065339791-N	C	T	T	T	T	T	T	T	T	T	C	C	C	C	C	C	C	C
13-084819885-M	G	A	A	A	A	A	A	A	A	A	G	G	G	G	G	G	G	G
13-101929792-M	C	T	T	T	T	T	T	T	T	T	C	C	C	C	C	C	C	C
13-117094028-M	G	A	A	A	A	A	A	A	A	A	G	G	G	G	G	G	G	G
14-005055006-M	T	A	A	A	A	A	A	A	A	A	T	T	T	T	T	T	T	T
14-024063411-M	A	G	G	G	G	G	G	G	G	G	A	A	A	A	A	A	A	A
14-040017031-M	G	C	C	C	C	C	C	C	C	C	G	G	G	G	G	G	G	G
14-060626617-N	T	C	C	C	C	C	C	C	C	C	T	T	T	T	T	T	T	T
14-077156430-M	C	T	T	T	T	T	T	T	T	T	C	C	C	C	C	C	C	C
14-097042967-M	A	G	G	G	G	G	G	G	G	G	A	A	A	A	A	A	A	A
14-114403652-M	C	T	T	T	T	T	T	T	T	T	C	C	C	C	C	C	C	C
15-003094890-M	A	G	G	G	G	G	G	G	G	G	A	A	A	A	A	A	A	A
15-020280219-M	G	T	T	T	T	T	T	T	T	T	G	G	G	G	G	G	G	G
15-043015319-M	G	C	C	C	C	C	C	C	C	C	G	G	G	G	G	G	G	G
15-059754938-N	C	T	T	T	T	T	T	T	T	T	C	C	C	C	C	C	C	C
15-102788257-N	G	T	T	T	T	T	T	T	T	T	G	G	G	G	G	G	G	G
16-005053446-C	G	T	T	T	T	T	T	T	T	T	G	G	G	G	G	G	G	G
16-019621494-C	A	G	G	G	G	G	G	G	G	G	A	A	A	A	A	A	A	A
16-057482753-C	A	G	G	G	G	G	G	G	G	G	A	A	A	A	G	G	G	G
16-075684315-C	T	C	C	C	C	C	C	C	C	C	T	T	T	T	T	T	T	T
16-096070057-C	C	T	T	T	T	T	T	T	T	T	C	C	C	C	C	C	C	C
17-003335010-M	G	C	C	C	C	C	C	C	C	C	G	G	G	G	G	G	G	G
17-023157746-M	A	T	T	T	T	T	T	T	T	T	A	A	A	A	A	A	A	A
17-043164647-M	G	A	A	A	A	A	A	A	A	A	G	G	G	G	G	G	G	G
17-066532305-N	T	G	G	G	G	G	G	G	G	G	T	T	T	T	T	T	T	T
17-083294998-M	T	A	A	A	A	A	A	A	A	A	T	T	T	T	T	T	T	T
17-092673068-N	T	G	G	G	G	G	G	G	G	G	T	T	T	T	T	T	T	T
18-005066417-M	T	C	C	C	C	C	C	C	C	C	T	T	T	T	T	T	T	T
18-022053385-M	A	G	G	G	G	G	G	G	G	G	A	A	A	A	A	A	A	A
18-043081750-M	C	T	T	T	T	T	T	T	T	T	C		C	C	C	C	C	C
18-054835717-M	T	A	A	A	A	A	A	A	A	A	T	T	T	T	T	T	T	T
18-067972780-M	T	C	C	C	C	C	C	C	C	C	T	T	T	T	T	T	T	T
18-078813035-M	A	C	C	C	C	C	C	C	C	C	A	A	A	A	A	A	A	A
18-090060367-M	G	T	T	T	T	T	T	T	T	T	G	G	G	G	G	G	G	G
19-018239318-M	T	G	G	G	G	G	G	G	G	G	T	T	T	T	T	T	T	T
19-021081804-M	A	G	G	G	G	G	G	G	G	G	A	A	A	A	A	A	A	A
19-038092479-M	T	C	C	C	C	C	C	C	C	C	T	T	T	T	T	T	T	T
19-048069669-N	C	T	T	T	T	T	T	T	T	T	C	C	C	C	C	C	C	C
19-060823449-N	A	G	G	G	G	G	G	G	G	G	A	A	A	A	A	A	A	A
X-016114535-G	C	A	A	A	A	A	A	A	A	A	C	C	C	C	C	C	C	C
X-025132696-G	C	G	G	G	G	G	G	G	G	G	C	C	C	C	C	C	C	C
X-054650362-N	T	A	A	A	A	A	A	A	A	A	T	T	T	T	T	T	T	T
X-068494838-M	C	T	T	T	T	T	T	T	T	T	C	C	C	C	C	C	C	C
X-087817653-G	A	T	T	T	T	T	T	T	T	T	A	A	A	A	A	A	A	A

X-100413449-M	T	G	G	G	G	G	G	G	G	G	T	T	T	T	T	T	T	T
X-114340727-M	A	G	G	G	G	G	G	G	G	G	A	A	A	A	A	A	A	A
X-133535206-G	T	C	C	C	C	C	C	C	C	C	T	T	T	T	T	T	T	T
X-143466659-M	A	C	C	C	C	C	C	C	C	C	A	A	A	A	A	A	A	A
